# The effects of fermentation of *Qu* on the digestibility and structure of waxy maize starch

**DOI:** 10.3389/fpls.2022.984795

**Published:** 2022-08-16

**Authors:** Wenhao Wu, Xudong Zhang, Jianzhou Qu, Renyuan Xu, Na Liu, Chuanhao Zhu, Huanhuan Li, Xingxun Liu, Yuyue Zhong, Dongwei Guo

**Affiliations:** ^1^Key Laboratory of Biology and Genetic Improvement of Maize in Arid Area of Northwest Region, College of Agronomy, Northwest A&F University, Yangling, China; ^2^Institute of Crop Science, Quality of Plant Products, University of Hohenheim, Stuttgart, Germany; ^3^Key Laboratory of Grains and Oils Quality Control and Processing, Collaborative Innovation Center for Modern Grain Circulation and Safety, College of Food Science and Engineering, Nanjing University of Finance and Economics, Nanjing, China; ^4^Department of Plant and Environmental Sciences, Faculty of Science, University of Copenhagen, Copenhagen, Denmark

**Keywords:** waxy maize starch, fermentation, *Qu*, starch digestibility, resistant starch

## Abstract

The fermentation of *Qu* (FQ) could efficiently produce enzymatically modified starch at a low cost. However, it is poorly understood that how FQ influences the waxy maize starch (WMS) structure and the digestion behavior. In this study, WMS was fermented by *Qu* at different time and starches were isolated at each time point, and its physico-chemical properties and structural parameters were determined. Results showed that the resistant starch (RS), amylose content (AC), the average particle size [D(4,3)] the ratio of peaks at 1,022/995 cm^–1^, and the onset temperature of gelatinization (T_*o*_) were increased significantly after 36 h. Conversely, the crystallinity, the values of peak viscosity (PV), breakdown (BD), gelatinization enthalpy (ΔH), and the phase transition temperature range (ΔT) were declined significantly after 36 h. It is noteworthy that smaller starch granules were appeared at 36 h, with wrinkles on the surface, and the particle size distribution was also changed from one sharp peak to bimodal. We suggested that the formation of smaller rearranged starch granules was the main reason for the pronounced increase of RS during the FQ process.

## Introduction

Starch is a primary energy source in the daily diets of humans (Meng et al., 2020). According to the enzymatic hydrolysis rate *in vitro*, starch can be classified as rapidly digested starch (RDS) (digested within 20 min), slowly digested starch (SDS) (digested between 20 and 120 min), and resistant starch (RS) (not digested within 120 min) (Englyst et al., 1992). This digestion pattern exists in both animals and humans, where RDS and SDS digestions are localized to the small intestine, but RS can only be fermented into short-chain fatty acids in the large intestine (Duyen et al., 2020). The *in vivo* clinical experiments indicate that the digestion rate of starch in the human body is closely related to the postprandial blood glucose response. Starch with high RDS will cause the human body to be in a state of hyperglycemia for a long time and induce the occurrence of chronic diseases, such as type II diabetes, obesity, and cardiovascular diseases (Lal et al., 2021a). Starch that is abundant with SDS can effectively postpone the increment of blood glucose and reduce chronic diseases. The short-chain fatty acids derived from RS ferment can effectively prevent the occurrence of colon cancer (He et al., 2021). Therefore, improving the content of SDS and RS in starch is of great significance for human nutrition and health.

There are many methods to modify the digestibility of starch, such as physical, chemical, and enzymatic methods. The physical methods change the multi-scale structure of starch through hydrothermal treatment, high temperature, or high pressure, which causes the rearrangement of the starch structure and reduces the digestibility of starch (Lal et al., 2021b). However, target-guided physical modification of starch is difficult since the multi-scale structure is hard to control, and thus, the production efficiency is relatively low. Chemical modification methods mitigate the digestibility of starch by reducing the specific binding between amylase and starch or changing the structure of starch by introducing new functional groups, such as oxidation, esterification, and etherification (Wolf et al., 1999). However, these inserted functional groups may bring potential food safety risks. The enzyme method is also reported to be an efficient way to produce modified starch because substrates can specifically combine with active sites of enzymes (Dura et al., 2014). Given that enzyme types, doses, or digestion times diversify, the starch structure can be differentially modified to some extent. Under the action of 1% isoamylase for 24 h, the content of RS was increased from 0.4 to 67.7% and 4.3 to 68% in waxy wheat and corn, respectively (Cai and Shi, 2010). The RS content of red kidney bean starch was increased from 21.3 to 31.5% by using pullulanase for 10 h (Reddy et al., 2013). However, the high cost of enzymatic modification limits its large-scale production.

Since ancient times in China, *Qu* has been widely used to produce wine at a low cost (Chen and Xu, 2013). Wine production by the fermentation of *Qu* (FQ) is a process in which a large number of glucoamylases and amylases are produced, and starches are degraded into glucose (Zhang et al., 2016). Then, yeast hydrolyzes glucose into alcohol and carbon dioxide during the anaerobic fermentation process (Mo et al., 2010). In this process, amylase only acts on α-1,4 glycosidic bonds, whereas glucoamylase not only acts on α-1,6 glycosidic bonds but also attacks on α-1,4 glycosidic bonds (Keeratiburana et al., 2020). There are also a large number of residues after the process of brewing, named vinasse, which is rich in starches. Studies have shown that the content of SDS in glutinous rice vinasse increased significantly after fermentation (Zhang et al., 2016), and the digestibility of different types of rice varies during fermentation, which may be related to their own structure (Situ et al., 2019). Therefore, the process of FQ can effectively change the structure of starch at a low cost.

Waxy maize is rich in nutrients, such as amino acids, protein, and vitamins, which are healthful for humans (Gong et al., 2018), and it is mainly used as fresh-eating maize (Ketthaisong et al., 2014). In addition, waxy maize is considered as a better material for the brewing of wine because of its higher ethanol conversion efficiency (Yangcheng et al., 2013). However, there is little knowledge on the starches in waxy maize vinasse, which limited the application of these starches. Therefore, we hypothesize that the FQ affected the digestibility and structures of waxy maize starch (WMS). To gain a broad insight into the consequences of FQ on WMS, we focused on the waxy maize for different fermentation times and explored the change of digestibility and structures of WMS at each time point.

## Materials and methods

### Materials

Waxy maize starch (WMS, Shan Bainuo192) was obtained from Maize Genetic Breeding Laboratory at Northwest A&F University in China. Pancreatin (Cat. No. P7545, Sigma, St. Louis, MO, United States) and amyloglucosidase (Cat. No. A7095, Sigma, St. Louis, MO, United States) were obtained from Sigma (St. Louis, MO, United States). *Qu* was purchased from Angel Yeast Co., Ltd. (Yichang, Hubei Province, China). The GOPOD Kit was purchased from Megazyme (Bray Business Park, Bray, Co., Wicklow, Ireland). All other chemical reagents were analytical grade.

### The fermentation of *Qu* and starch isolation

The maize flour (100 g) was mixed evenly with both water and *Qu* in a sealed tank. Maize flour, water, and *Qu* were in the proportion of 250:75:1 according to the instructions of the manufacturer, and the suspension was fermented at a 30°C constant temperature incubator for 12, 24, 36, 48, and 60 h, respectively. Maize vinasse at each time point was collected to extract starch (Zhang et al., 2016).

Starch was isolated according to the previous methods with some modifications (Lin et al., 2016). Maize vinasse was ground with water and filtered through a 100 μm sieve into a 50 ml centrifuge tube. This suspension was centrifuged at 1,500 rpm and room temperature for 8 min, and the supernatant was discarded. Sodium hydroxide (0.4%, W/V) was added to the precipitate and shaken well. This suspension was incubated for 4 h and then centrifuged at 1,500 rpm for 4 min, and the supernatant was discarded. The precipitate was washed three times with distilled water and centrifuged at 1,500 rpm for 4 min. Finally, the precipitate was washed with ethanol and centrifuged at 4,000 rpm for 10 min. The precipitate was air-dried, and then fermented maize starch was obtained.

### Amylose content

The measurement of amylose content (AC) was conducted according to the dual wavelength method (Zhu et al., 2008). Potassium iodide (KI) (2 g) and iodine (I_2_) (0.2 g) were dissolved in 100 ml of deionized water to obtain iodine solution. Starch (10 mg) was dispersed in 100 μl of ethanol. Sodium hydroxide solution (1 M, 1 ml) and deionized water (8.9 ml) were added to the suspension to form Solution A. Then, Solution A (200 μl), 0.1 M HCl (200 μl), iodine solution (200 μl), and deionized water (9.4 ml) were mixed evenly. The absorbance of this solution at 510 and 620 nm was detected by UV spectrophotometry (Shimadzu, Japan). The AC was calculated according to the following formula:


Amylose=0.95×[(ABS-620ABS)510+0.0542]/0.3995

### Scanning electron microscopy

The morphology of starch granules was observed by SEM (S-3400N, Hitachi, Japan) at a voltage of 5 kV. Samples were sprayed with gold before observation (Song et al., 2019).

### Laser diffraction particle size

The particle size distributions of starch granules were analyzed by Laser diffraction particle size (LDPS) analyzer (Hydyo2000MU, Mastersizer 2000, United Kingdom) with a resolution of 0.1–1,000 μm (Song et al., 2019).

### X-ray diffraction

Crystal patterns of starch granules were analyzed by X-ray diffraction (XRD) (D/max2200pc, Rigaku, Japan). The X-ray beam was set to 40 mA and 40 kV, the diffraction angle range (2θ) was 3–40°, and the scanning speed was 4°/min. The relative crystallinity (RC) was calculated by MDI JADE 6.0 software (Song et al., 2019).

### Fourier transform infrared spectroscopy

The short-range ordered structure of starch was determined by Fourier transform infrared (FTIR) spectroscopy (Nicolet IS50, Thermo Fisher Scientific, Hudson, United States). The wavelength range of 800–1,200 cm^–1^ was applied, and OMNIC software (Thermo Fisher Scientific, United States) was used for deconvolution. The peak width was 70.4 cm^–1^, and the resolution enhancement factor was 2.0 (Song et al., 2019).

### Differential scanning calorimetry

The thermal properties of starch were detected by differential scanning calorimetry (DSC, Q2000, TA Instruments, Waters, United States). Starch (3 mg) and deionized water (9 μl) were mixed evenly in a sealed aluminum container and incubated overnight to achieve hydration. Moreover, a blank aluminum container was used as a blank control. The scanning range was 30–120°C, and the scanning speed was 10°C/min. The onset gelatinization temperature (T_*o*_), peak temperature (T_*p*_), conclusion temperature (T_*c*_), and gelatinization enthalpy (ΔH) were determined (Song et al., 2019).

### Pasting property analysis *via* rapid viscosity analysis

The pasting properties of starch were measured by a rapid viscosity analyzer (RVA-4500, Perten Instruments, Sweden). Starch (3 g) was dissolved in 25 ml of deionized water. The starch suspension was first heated at 50°C for 1 min, heated to 95°C for 2.5 min at a rate of 12°C/min, and finally cooled to 50°C for 2 min at the same rate. The peak viscosity (PV), trough viscosity (TV), final viscosity (FV), breakdown (BD), and setback (SB) values were measured (Song et al., 2019).

### *In vitro* digestibility

The digestibility of starch was evaluated according to the Englyst method (Englyst et al., 1992) with slight modifications. The enzyme solution was prepared by mixing 1,160 mg porcine pancreatin (Cat. No. P7545, Sigma, St. Louis, MO, United States), 692 μl of amyloglucosidase (Cat. No. A7095, Sigma, St. Louis, MO, United States), and 6 ml of sodium acetate buffer (0.1 M, pH = 5.2). Starch (100 mg), sodium acetate buffer (15 ml), and enzyme solution (2.5 ml) were shaken well in a 50 ml centrifuge tube. This suspension was incubated in a shaker at 37°C at 300 rpm. The 0.1 ml reacted solution was collected at different time points (0, 10, 20, 30, 40, 60, 90, 120, and 180 min), and the enzyme was inactivated by adding 1 ml of anhydrous ethanol. This suspension was centrifuged at 10,000 rpm for 5 min, and the glucose content of the supernatant was determined by a GOPOD Kit. The contents of RDS (the starch digested within 0–20 min), SDS (the starch digested within 20–120 min), and RS (the remaining residue) were calculated as described before (Zhong et al., 2022).

### Statistical analysis

All experiments were conducted with three biological replicates. The data are expressed as the mean ± standard deviation (SD). SPSS 22 was used to perform a one-way analysis of variance (ANOVA) and least significant difference (LSD) tests (*p* < 0.05) to determine whether there were significant differences among the results.

The abbreviation is exemplified as “starch type-fermentation time.” For instance, “WMS-12” indicates that the WMS was fermented for 12 h.

## Results

### Effects of fermentation of *Qu* on starch digestion pattern

The content of RDS was almost not changed within 36 h and significantly decreased after 36 h, the minimum value (12.4%) was obtained at 60 h. In contrast, the content of RS was increased during the process of fermentation, and the predominant increase happened after 36 h, the maximum value (65.6%) was obtained at 60 h. As for SDS, it did not change within 24 h and then decreased significantly, and the minimum value (22.0%) was also obtained at 60 h (Table 1). These results indicated that the digestibility of WMS was mitigated after FQ.

**TABLE 1:** *In vitro* digestion profiles of starches from native and fermented starch.

Samples	RDS (%)	SDS (%)	RS (%)
WMS	28.0 ± 1.1^ab^	72.0 ± 1.1^a^	0.0 ± 0.0^b^
WMS-12	29.1 ± 0.9^ab^	70.9 ± 0.9^ac^	0.0 ± 0.0^b^
WMS-24	21.5 ± 0.3^ab^	74.3 ± 2.2^a^	4.2 ± 1.9^b^
WMS-36	33.1 ± 10.0^a^	54.59 ± 6.1^bc^	12.3 ± 3.9^b^
WMS-48	14.4 ± 5.3^b^	33.5 ± 6.0^d^	52.1 ± 7.2^a^
WMS-60	12.4 ± 3.0^b^	22.0 ± 5.6^d^	65.6 ± 2.6^a^

All data are means ± standard deviations (SD). Values with different letters in the same column are significantly different at p < 0.05. RDS, rapidly digestible starch; SDS, slowly digestible starch; RS, resistant starch; WMS, native waxy maize starch; WMS-X, waxy maize starch fermented for X h.

### Effects of fermentation of *Qu* on amylose content of starch

The AC of WMS was increased with the increasing fermentation time, and it significantly increased after 36 h (Table 2). A possible explanation is that the debranching of amylopectin under the effects of glucoamylase and the long-branch chains could be recognized as amylose (Liu et al., 2019).

**TABLE 2:** The amylose content, crystallinity, Fourier transform infrared (FTIR), and particle size of native and fermented starch.

Samples	AC (%)	RC (%)	1,047/1,022 cm^–1^	1,022/995 cm^–1^	D(4,3)
WMS	6.9 ± 0.2 ^c^	52.5 ± 2.2^a^	0.9 ± 0.0^a^	0.7 ± 0.1^b^	26.0 ± 0.1^d^
WMS-12	7.7 ± 0.8^bc^	47.7 ± 2.2^a^	1.0 ± 0.3^a^	0.8 ± 0.1^b^	26.6 ± 0.9^d^
WMS-24	9.0 ± 0.9^bc^	38.8 ± 1.4^b^	1.2 ± 0.0^a^	0.8 ± 0.1^b^	25.0 ± 0.7^d^
WMS-36	9.9 ± 0.1^b^	33.6 ± 2.2^b^	1.2 ± 0.0^a^	1.7 ± 0.5^a^	28.8 ± 0.7^c^
WMS-48	13.0 ± 1.5^a^	9.6 ± 0.5^c^	1.1 ± 0.0^a^	1.4 ± 0.1^a^	39.2 ± 0.1^b^
WMS-60	14.0 ± 0.5^a^	8.0 ± 0.1^c^	1.0 ± 0.0^a^	1.5 ± 0.1^a^	48.0 ± 1.0^a^

All data are means ± standard deviation (SD). Values with different letters in the same column are significantly different at p < 0.05. AC, amylose content; RC, relative crystallinity; 1,047/1,022, the ratio of peaks at 1,047/1,022 cm^–1^; 1,022/995, the ratio of peaks at 1,022/995 cm^–1^; D(4,3), mean diameter over the surface distribution. WMS, native waxy maize starch; WMS-X, waxy maize starch fermented for X h.

### Effects of fermentation of *Qu* on the morphology of starch

**FIGURE 1:**
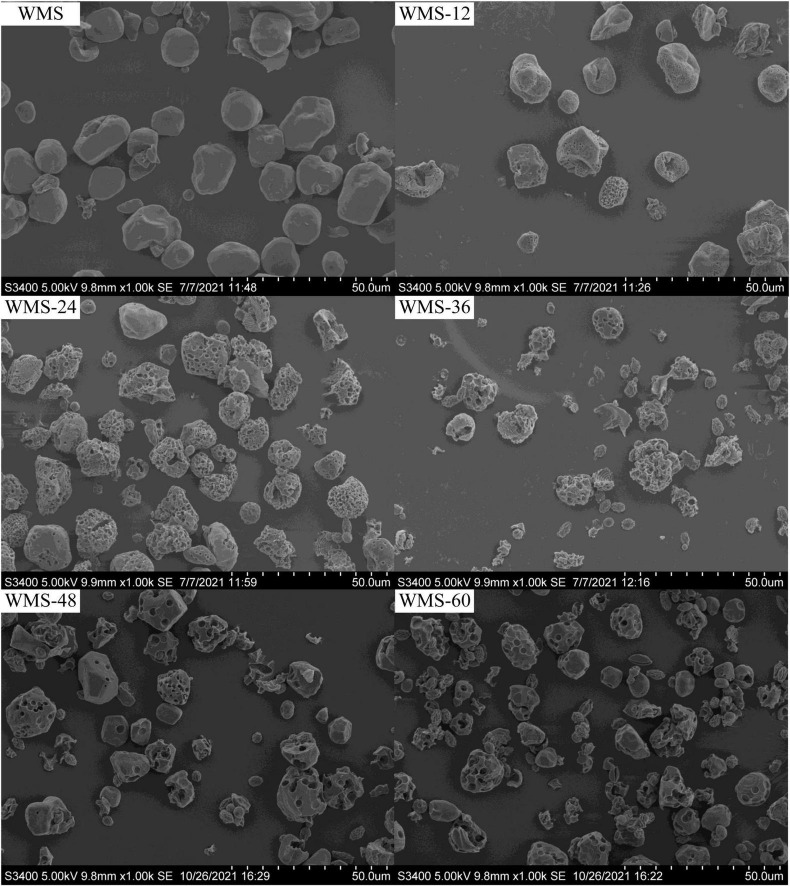
Scanning electron microscopy images showing the morphology of native starch and fermented starch. WMS, native waxy maize starch fermented for x h.

The shape of native WMS was irregular polygon, consistent with previous research results (Chen et al., 2006). During the fermentation, a number of pores began to appear on the surface and their number and diameter were increased gradually with the extension of fermentation time. It is noteworthy that some starch particles were completely destroyed, and smaller particles with wrinkles on the surface appeared at 36 h; the number also increased gradually with the extension of fermentation time (Figure 1).

### Effects of fermentation of *Qu* on the particle size distribution of starch

**FIGURE 2:**
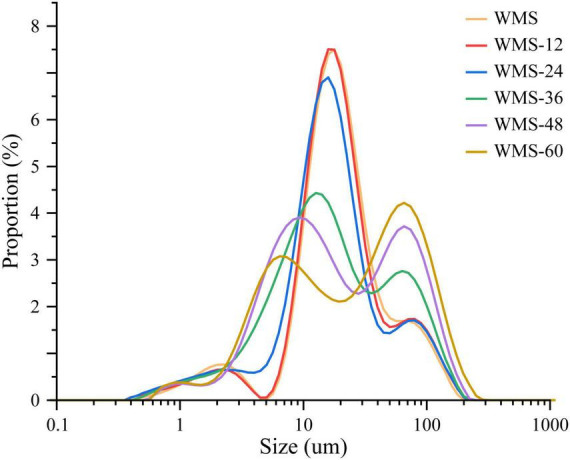
The particle size distribution of native starch and fermented starch. WMS, native waxy maize starch; WMS-x, waxy maize starch fermented for x h.

The fermentation did not change the average granular size (D [4, 3]) of WMS within 24 h and was significantly increased after 24 h (Table 2). This may be due to the small granules that were attacked by enzymes preferentially, because it could provide more effective binding sites for enzymes (Warren et al., 2011). It is also noteworthy that there appeared two sharp peaks at 36 h, the first peak was dropped and transferred toward the left gradually and the second peak was only increased with the extension of fermentation time (Figure 2). It means that the proportion of large granule was increased and the smaller particles were appeared. This result was also according to the SEM (Figure 1).

### Effects of fermentation of *Qu* on crystalline structures of starch

**FIGURE 3:**
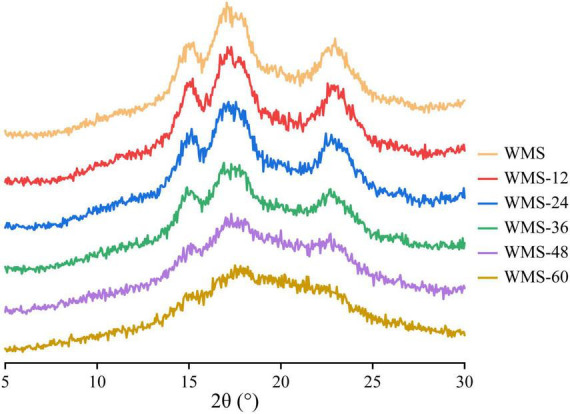
X-ray diffraction spectra of native starch and fermented starch. WMS, native waxy maize starch; WMS-x, waxy maize starch fermented for x h.

The native WMS had strong diffraction peaks at 15, 17, 18, and 23° (Figure 3), which is a typical A-type crystal structure (Qiao et al., 2016). Moreover, the fermentation process did not change the crystal type of starch, consistent with previous results (Zhang et al., 2016). However, the diffraction peaks were almost disappeared after 36 h (Figure 3). It means that the crystal structure of WMS was destroyed severely. This is also confirmed by the crystallinity data, which were always decreased during fermentation and the pronouncedly decreasing happened after 36 h, and the minimum value (8.04%) was obtained at 60 h (Table 2).

### Effects of fermentation of *Qu* on the short-range ordered structure of starch

**FIGURE 4:**
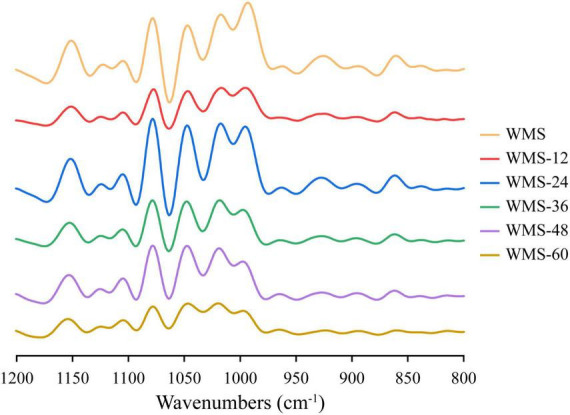
Fourier transform infrared spectra of native starch and fermented starch. WMS, native waxy maize starch; WMS-x, waxy maize starch fermented for x h.

Compared to native WMS, the FQ did not change the FTIR spectrum of starch (Figure 4). The peak intensities of starch at 1,047 and 1,022 cm^–1^ are representative of the crystalline and amorphous regions, respectively (Wang et al., 2016). Therefore, the ratio of peak intensity at 1,047/1,022 cm^–1^ and 1,022/995 cm^–1^, respectively, could be used to reflect the degree of order in starch and the proportion of amorphous to order structure of starch (Cai et al., 2014). The ratio of 1,047/1,022 cm^–1^ was increased within 36 h and then decreased, and the difference was insignificant (Table 2), which indicated that the structure of WMS became more order within 36 h. While the ratio of 1,022/995 cm^–1^ was increased within 36 h and then obtained a plateau. It demonstrated that a more amorphous region appeared at 36 h during fermentation.

### Effects of fermentation of *Qu* on the thermal properties of starch

The onset gelatinization temperature (T_*o*_) of starch did not change within 36 h. However, it increased pronouncedly after 36 h. The peak gelatinization temperature (T_*p*_) showed a similar trend, which was increased significantly after 36 h, and the change within 36 h can be ignored. As for the conclusion gelatinization temperature (T_*c*_), it was raised within 24 h and then dropped, and the maximum value was obtained at 24 h. Both the gelatinization enthalpy (ΔH) and the transition temperature range (ΔT) showed a similar trend, which did not change within 36 h and then was decreased significantly (Table 3).

**TABLE 3:** The thermal properties of native and fermented starch.

Samples	T_*o*_ (°C)	T_*p*_ (°C)	T_*c*_ (°C)	Δ T (°C)	Δ H (J/g)
WMS	65.4 ± 0.3^c^	70.1 ± 0.0^c^	79.9 ± 0.7^bc^	14.4 ± 0.9^bc^	7.6 ± 0.3^ab^
WMS-12	66.0 ± 0.6^bc^	71.0 ± 0.1^b^	81.2 ± 0.0^ab^	15.2 ± 0.6^abc^	9.2 ± 1.1^a^
WMS-24	65.8 ± 0.2^c^	71.1 ± 0.3^ab^	82.2 ± 0.9^a^	16.5 ± 0.7^a^	9.2 ± 0.4^a^
WMS-36	65.2 ± 0.0^c^	70.1 ± 0.0^c^	81.0 ± 0.0^ab^	15.8 ± 0.0^ab^	6.5 ± 0.0^b^
WMS-48	67.0 ± 0.4^ab^	71.9 ± 0.5^a^	80.8 ± 0.2^ab^	13.8 ± 0.2^c^	4.0 ± 0.4^c^
WMS-60	67.7 ± 0.0^a^	71.9 ± 0.1^a^	79.0 ± 0.1^c^	11.3 ± 0.1^d^	1.5 ± 0.3^d^

All data are means ± standard deviation (SD). Values with different letters in the same column are significantly different at p < 0.05. T_o_, the gelatinization onset temperature; T_p_, the gelatinization peak temperature; T_c_, the gelatinization final temperature; ΔT, T_c_ − T_o;_ ΔH: gelatinization enthalpy; WMS, native waxy maize starch; WMS-X, waxy maize starch fermented for X h.

### Effects of fermentation of *Qu* on pasting properties of starch

Fermentation of Qu decreased the pasting properties of WMS. The PV was declined continuously during fermentation, and it was significantly decreased after 36 h. Both the TV and BD showed a similar trend with PV, whereas the serious decline happened after 24 and 36 h, respectively, and then both were steadied. The changes of both FV and SB values had fluctuated, and they were always lower than the native starch, and the minimum values of them were obtained at 48 h (Table 4).

**TABLE 4:** The pasting properties of native and fermented starch.

Samples	PV(cP)	TV(cP)	BD(cP)	FV(cP)	SB(cP)
WMS	4539 ± 3^a^	1781 ± 63^a^	2772 ± 74^a^	2458 ± 9^a^	677 ± 71^a^
WMS-12	4016 ± 51^b^	1422 ± 18^b^	2594 ± 69^a^	2015 ± 20^b^	593 ± 38^a^
WMS-24	3243 ± 16^c^	1308 ± 27^b^	1935 ± 11^b^	1604 ± 25^c^	296 ± 3^b^
WMS-36	2042 ± 173^d^	873 ± 49^c^	1169 ± 124^c^	1141 ± 79^d^	268 ± 30^b^
WMS-48	1458 ± 4^e^	748 ± 2^c^	710 ± 2^d^	1014 ± 3^d^	266 ± 5^b^
WMS-60	1848 ± 153^d^	879 ± 31^c^	969 ± 122^cd^	1499 ± 87^c^	620 ± 56^a^

All data are means ± standard deviation (SD). Values with different letters in the same column are significantly different at p < 0.05. PV, peak viscosity; TV, trough viscosity; BD, breakdown; FV, final viscosity; SB, setback; WMS, native waxy maize starch; WMS-X, waxy maize starch fermented for X h.

## Discussion

Amylase and glucoamylase are mainly produced during the FQ (Zhang et al., 2016). Amylase randomly hydrolyzes α-1,4 glycosidic bonds, and glucoamylase can not only hydrolyze α-1,4 glycosidic bonds but also hydrolyze α-1,6 glycosidic bonds (Keeratiburana et al., 2020). Therefore, the essence of FQ is the enzymatic hydrolysis of starch by amylase and glucoamylase.

### First stage of fermentation of *Qu*: The formation of a more perfect starch structure

The increase of RS of WMS may be due to the structural changes under the action of enzymes during the FQ. The native WMS had a typical A-type crystallinity (Figure 3), and shorter branch chains (Hizukuri, 1985) and its branching points exist not only in amorphous regions but also in crystalline regions. Those led to a large number of “weak points” in the crystalline structure that can be easily hydrolyzed (Jane et al., 1997).

During FQ, the crystal structure was hydrolyzed partly within 36 h, as shown in the data of XRD, the crystallinity only decreased from 52.47 to 33.85% (Table 2), and the pattern of diffraction peaks did not change (Figure 3). The crystalline region is constituted of amylopectin (Perez and Bertoft, 2010). It was clear that the de-branching of long-branch chains of amylopectin could increase the AC (Liu et al., 2019). Our data showed that the content of amylose did not increase significantly within 36 h (Table 2), it demonstrated that the short-chains of amylopectin were hydrolyzed preferentially, which could lead to a more perfect structure of starch (Zhu, 2018). Because the short-chains of amylopectin could lead to form a number of “weak points” in the crystal structure, thus making it more sensitive to enzymes (Jane et al., 1997). The increase in the ratio of 1,047/1,022 cm^–1^ within 36 h also demonstrated this (Table 2; Zhang et al., 2010).

Amylopectin contributes to water absorption and starch swelling, while amylose inhibits this swelling (Tester and Morrison, 1990). This swelling property can be reflected by PV (Zou et al., 2019), the decrease in PV may be due to the loss of branch chains of amylopectin (Li et al., 2017). Therefore, we suggest that the decrease in PV within 36 h was mainly due to the hydrolysis of amylopectin, and the increase of AC (Table 2) was the reason for the decrease after 36 h. It is noteworthy noting that the SB did not show a positive correlation with AC, which is not according to the previous research (Li et al., 2016). We suggest that it is mainly due to the effects of the amylose length and the whole size of amylopectin (Tao et al., 2019), and we will explore it in a future study. The value of BD could be used to reflect the intermolecular force of starch. The smaller BD is, the stronger the intermolecular force is (Zou et al., 2019). Our data showed that both the PV and BD decreased pronouncedly within 36 h (Table 4), indicating that the amylopectin was destroyed and the intermolecular force of starch became stronger at that point, which indirectly indicated that the “weak points” were hydrolyzed and the crystal structure became more perfect. The RS content also increased from 0 to 10.1% (Table 1) within 36 h. Therefore, we suggested that the formation of a more perfect crystal structure was the reason for the increase in RS within 36 h.

### Second stage of fermentation of *Qu*: The formation of rearrangement starch granules

However, the crystal structure was almost destroyed completely at 60 h of FQ (Table 2). The diffraction peaks of XRD were almost disappeared after 36 h (Figure 3). In addition, the gelatinization enthalpy (ΔH) could indirectly reflect the crystal structure. The decrease in ΔH indicates that part of the double helix structures of starch was destroyed, and the energy required to destroy the double helix was relatively low (Qiao et al., 2016). Our data showed the ΔH decreased significantly after 36 h (Table 3), indicating that the crystal structure was destroyed seriously. The significant increase in AC after 36 h fermentation (Table 2) also indirectly demonstrated this. It is noteworthy that some smaller granules appeared with wrinkles on the surface (Figure 1), and the particle size distribution also changed from one sharp peak to bimodal (Figure 2). A possible reason is that the generation of short amylose and amylopectin chains under the action of enzymes could rearrange to form new starch granules (Ma et al., 2020) that included the formation of a double helix of amylose-amylose, amylose-amylopectin, and amylopectin-amylopectin (Li et al., 2015). In addition, these realignment granules had more intense structure, which is more enzyme resistant (Polesi and Sarmento, 2011).

Our data also demonstrated this, the value of BD was also decreased seriously at this time point, indicating the enhanced intermolecular force of starch. Meanwhile, the significant increase in gelatinization onset temperature (T_*o*_) and the pronounced narrowing of ΔT after 36 h (Table 3) also demonstrated the formation of a more intense starch structure (Ding et al., 2019). Therefore, we speculated that the formation of smaller rearranged starch granules was the main reason for the increase in RS during fermentation.

In conclusion, the effects of FQ on WMS may have two processes. Firstly, the “weak points” were hydrolyzed, which made the crystal structure become more perfect and the RS increased slightly (Table 1). Then, the crystal structure was almost hydrolyzed completely and could form smaller rearranged starch granules, which was the main reason for the pronounced increase in RS during the FQ.

## Conclusion

The RS content of WMS could be increased significantly by the FQ, which may be due to the crystal structure changed under the action of enzymes during fermentation. There may undergo two processes. Firstly, the “weak points” of the crystal structure were hydrolyzed preferentially, which made the crystal structure become more perfect and RS content increased slightly. In the second process, the crystal structure was almost destroyed completely, and the broken starch could be rearranged to form smaller starch granules, which was the main reason for the pronounced increase of RS during fermentation. Therefore, by controlling fermentation time, it could produce the WMS with higher RS content at a low cost, and it also could be applied to the functional food area on a large-scale in the future.

## Data availability statement

The original contributions presented in the study are included in the article/supplementary material, further inquiries can be directed to the corresponding authors.

## Author contributions

WW: conceptualization, visualization, investigation, writing-original draft, preparation, and writing-review and editing. XZ, NL, and XL: writing-review and editing. JQ: software. RX, CZ, and HL: investigation. YZ: conceptualization and writing-review and editing. DG: conceptualization, funding acquisition, and writing-review and editing. All authors contributed to the article and approved the submitted version.
